# Risk assessment and integrated surveillance of foot-and-mouth disease outbreaks in Russia based on Monte Carlo simulation

**DOI:** 10.1186/s12917-021-02967-x

**Published:** 2021-08-10

**Authors:** Jianying Wang, Jiahui Chen, Shuwen Zhang, Yanting Ding, Minjia Wang, Hui Zhang, Ruirui Liang, Qin Chen, Bing Niu

**Affiliations:** Shanghai Key Laboratory of Bio-Energy Crops, School of Life Sciences, Shanghai University, 200444 Shanghai, People’s Republic of China

**Keywords:** Foot-and-mouth disease (FMD), Monte Carlo simulations, Risk assessment, Defense measures, Spatial time scan statistic

## Abstract

**Background:**

Foot-and-mouth disease (FMD) is a highly contagious disease of livestock worldwide. Russia is a big agricultural country with a wide geographical area where FMD outbreaks have become an obstacle for the development of the animal and animal products trade. In this study, we aimed to assess the export risk of FMD from Russia.

**Results:**

After simulation by Monte Carlo, the results showed that the probability of cattle infected with FMD in the surveillance zone (Surrounding the areas where no epidemic disease has occurred within the prescribed time limit, the construction of buffer areas is called surveillance zone.) of Russia was 1.29 × 10^− 6^. The probability that at least one FMD positive case was exported from Russia per year in the surveillance zone was 6 %. The predicted number of positive cattle of the 39,530 - 50,576 exported from Russia per year was 0.06. A key node in the impact model was the probability of occurrence of FMD outbreaks in the Russian surveillance zone. By semi-quantitative model calculation, the risk probability of FMD defense system defects was 1.84 × 10^− 5^, indicating that there was a potential risk in the prevention and control measures of FMD in Russia. The spatial time scan model found that the most likely FMD cluster (*P* < 0.01) was in the Eastern and Siberian Central regions.

**Conclusions:**

There was a risk of FMDV among cattle exported from Russia, and the infection rate of cattle in the monitored area was the key factor. Understanding the export risk of FMD in Russia and relevant epidemic prevention measures will help policymakers to develop targeted surveillance plans.

**Supplementary Information:**

The online version contains supplementary material available at 10.1186/s12917-021-02967-x.

## Background

Foot-and-mouth disease (FMD) is a severe, highly contagious viral disease of livestock and wild cloven-hoofed animals worldwide. Cattle and swine, as well as sheep, goats, and other cloven-hoofed ruminants are domesticated species that can be infected easily. Wild cloven-hoofed animals, including deer, antelope, elephants and giraffes, are susceptible to be infected by FMD [1–3], which is characterized by fever and blister-like sores on the feet, mouth, nares, muzzle and teats [4] and does not result in high mortality in adult animals. However, the pain and discomfort from the lesions make the animals depressed, anorexic, lame and reluctant to move [5]. Foot-and-mouth disease virus (FMDV) is a non-enveloped RNA virus, belonging to the *Aphthovirus* genus of the *Picornaviridae* family [6], is divided into seven serotypes (A, O, C, Asia 1, SAT1, SAT2 and SAT3) based on serological results [7]. A non-structural protein (NSP) could be found in animals with viral proliferation and in the culture of infected cells, which can be used to distinguish between infected and uninfected animals, regardless of their vaccination status [8].

**Fig. 1:**
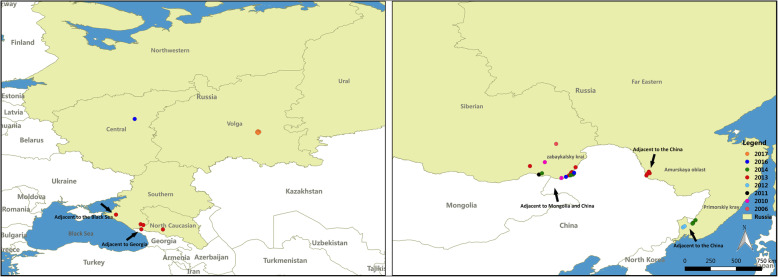
Map of Russia showing the outbreaks of FMD during 2006 ~ 2017. The maps were generated with QGIS Version 2.18 (https://www.qgis.org/en/site/index.html) and the base map was Bing Maps from QuickMapServices

The Russian Federation is the world’s largest land-area country [9, 10], with fertile land and excellent conditions for agricultural development. However, in recent years, livestock were seriously threatened by FMD [11]. FMD outbreaks have been largely concentrated on the Russian border. Since 2005, 55 outbreaks of FMD have been reported in Russia (18 Asia type 1 cases, 13 type O cases and 24 type A cases) [12]. The outbreaks from 2005 to 2017 were concentrated in the northern Caucasus region, adjacent to the Black sea, the Zabaykalsky Krai, the Amurskaya Oblast and the Primorskiy Kray, which is adjacent to Georgia, China and Mongolia (Fig. 1**)**. In 2014, four FMD outbreaks (including three cases of A-type and one case of O-type) occurred in the Trans-Baikal territory region, and 28 cattle were infected [13]. As one of Russia’s trading nations, China began importing beef from Russia in 2019 [14]. FMD frequently occurs in Russia with cattle especially calves most susceptible for FMD. Therefore, the cattle are at risk of being exposed to the virus and infection, which will bring losses to the livestock breeding industry of importing countries. Therefore, it is important to assess the risk of Russian cattle exports.

To evaluate the risk of animal disease transmission, stochastic decision trees were proposed to prevent disease. Stochastic decision trees are a risk analysis approach that provides accurate simulation results for future change. Pearson [15] and Hernandez-Jover et al. [16] used decision trees to represent exposure pathways in susceptible animals and calculated the probability of occurrence of these pathways using a Monte Carlo stochastic simulation model. Herrera-Ibatá et al. [17] summarized the event structure and event chain of the risk path of the legitimate import (Import animals and animal products according to international or national laws) of live pigs and swine products by constructing the decision tree. Finally, the high-risk time (year) and region of import were determined by simulation. In the field of FMD, stochastic decision tree combined with Monte Carlo simulations have been used in the quantitative assessment of the risk of FMD [18]. Some scientists applied stochastic decision trees and Monte Carlo stochastic modeling to investigate the risk of introduction and spread of FMD in sheep [19] and pigs [20]. However, these studies only analyzed the exposure probability and transmission scenario but did not carry out further research, such as the assessment of relevant epidemic prevention measures.

**Fig. 2:**
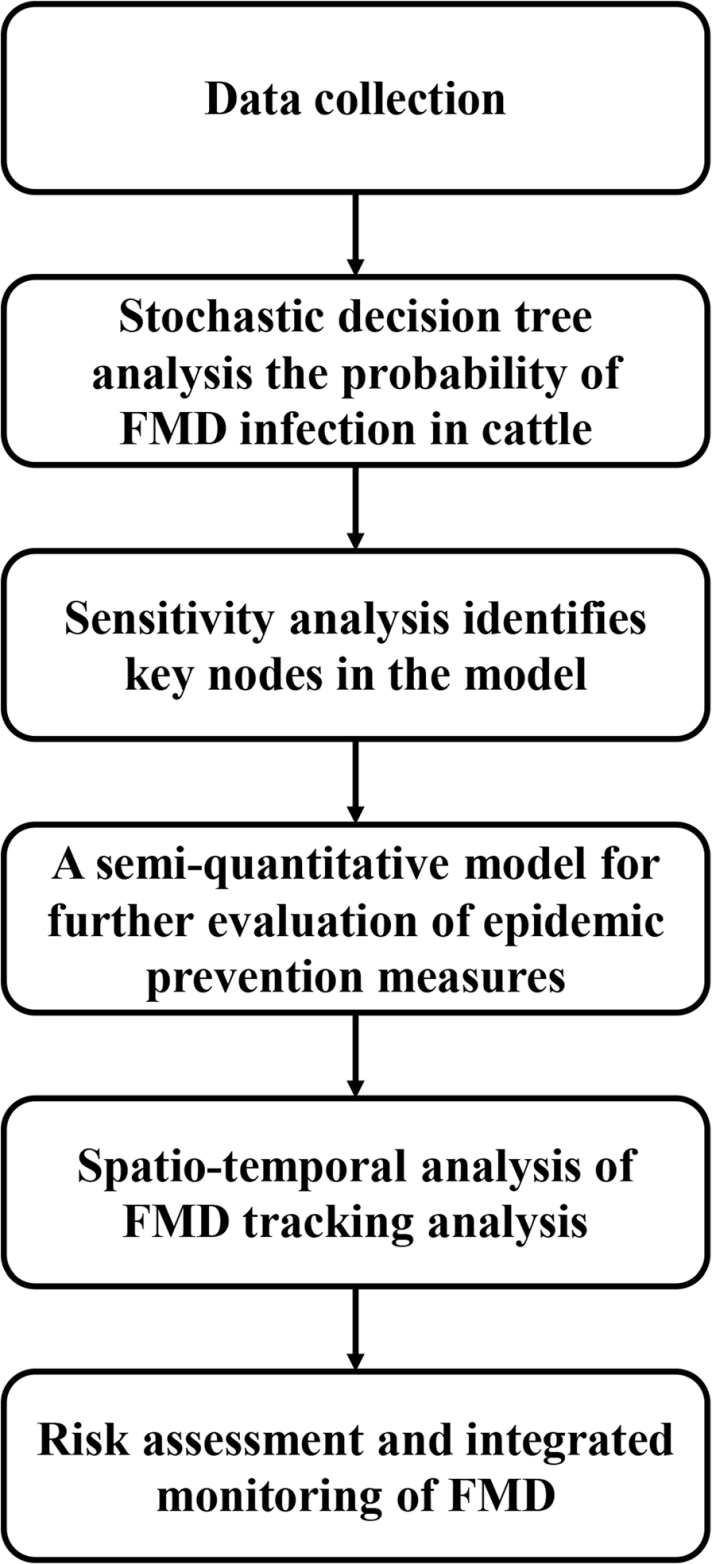
Flow chart in integrated surveillance of FMD outbreaks

In this study, we aimed to assess the export risk of FMD from Russia by combining quantitative risk analysis, semi-quantitative risk analysis and spatio-temporal scanning. First, the stochastic decision tree was used to build a quantitative risk assessment model. Then, risk assessment of epidemic prevention measures was carried out based on decision tree sensitivity analysis and semi-quantitative method. Finally, monitoring statistical data of spatio-temporal scanning was used to provide a basis for the division of regulatory areas (Fig. 2). The stochastic decision tree and semi-quantitative method are combined with a geographic information system for a more comprehensive assessment. We hope that our study will provide a theoretical basis for the formulation of prevention and control measures against FMD in Russian cattle, and provide a reference for the risk of FMD brought by China and other countries importing Russian cattle.

## Results

### Stochastic decision tree model to assess the risk for FMD outbreaks in the surveillance zone

A four-tiered risk stochastic decision tree distribution model was established to assess the risk of cattle being infected with FMD in surveillance zones in Russia. The sensitivity of the output variable to the input distribution was analyzed to determine the critical inputs in the model.

**Fig. 3:**
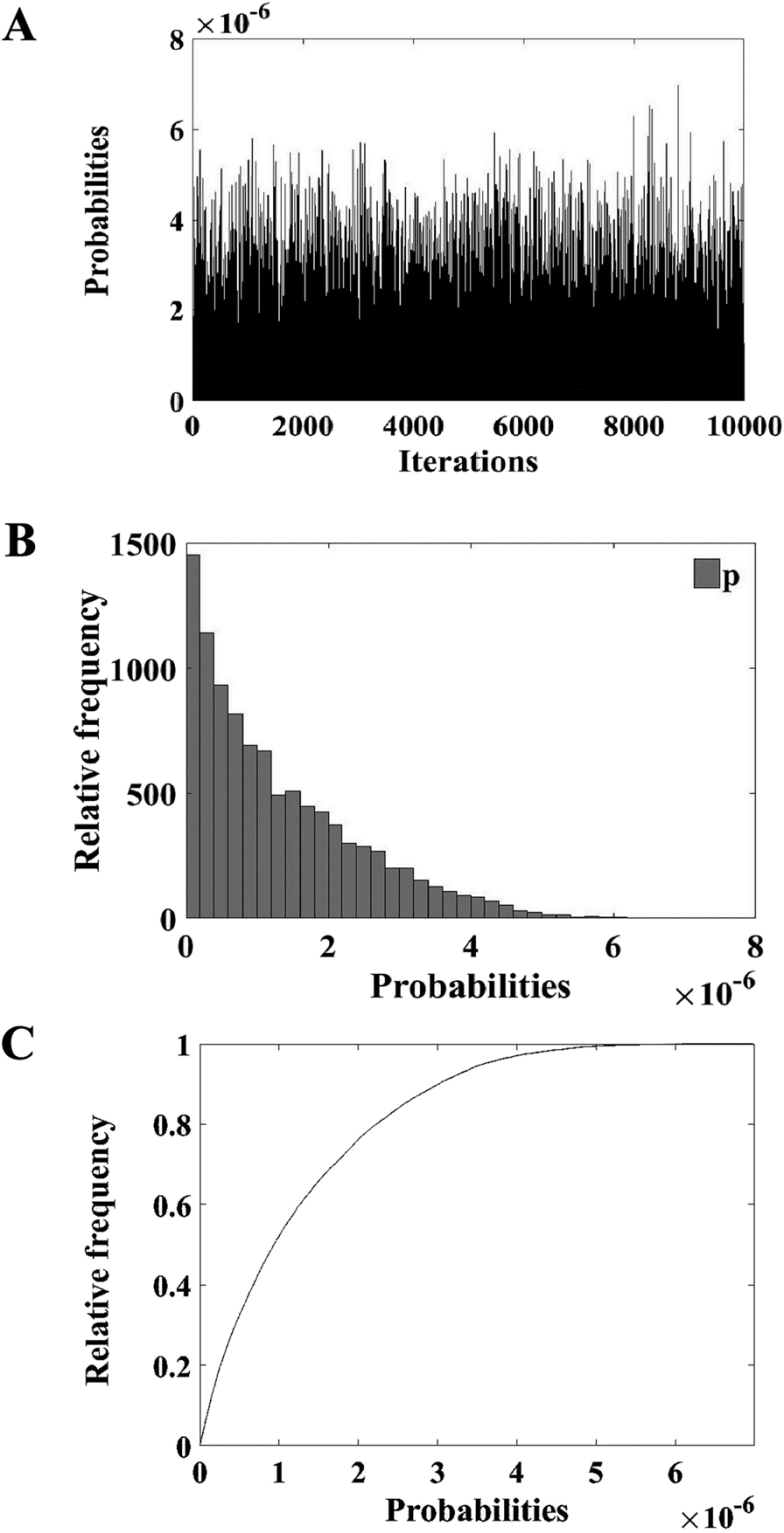
Output of model after 10,000 iterations. **A**: Probability distribution of 10,000 of model after 10,000 iterations, **B**: Relative frequency of model after 10,000 iterations, **C**: Cumulative frequency of model after 10,000 iterations

The values of the parameters for each node are shown in Table 1. After 10,000 iterations, the mean probability distribution of the model output was determined (Fig. 3). p: The probability of cattle infected with FMD in the surveillance zones of Russia was 1.29 × 10^− 6^, q: The probability of one FMD positive case exported from Russia per year was 6 %, e: The predicted number of positive cattle of the 39,530 − 50,576 exported from Russia per year was 0.06. The results of the model showed there was low risk in the cattle exported from Russia, and the risk could be further reduced by taking appropriate risk management measures (Table 2).

**Table 1:** Summary of nodes and parameters used in the modeling

Node	Distribution	Value	
		a/α1	b/α2
N	Uniform	39,530	50,576
P1	Uniform	0	0.333
P2	Beta	71	4380
P3	Beta	103	17,089
P4	Uniform	0.003	0.160

**Table 2:** Outputs for the risk of entry of FMD through importation of cattle from Russia

Outputs	Minimum	Mean	Maximum
p	5.97 × 10^− 12^	1.29 × 10^− 6^	7.65 × 10^− 6^
q	2.78 × 10^− 7^	0.06	0.32
e	2.78 × 10^− 7^	0.06	0.38

**Fig. 4:**
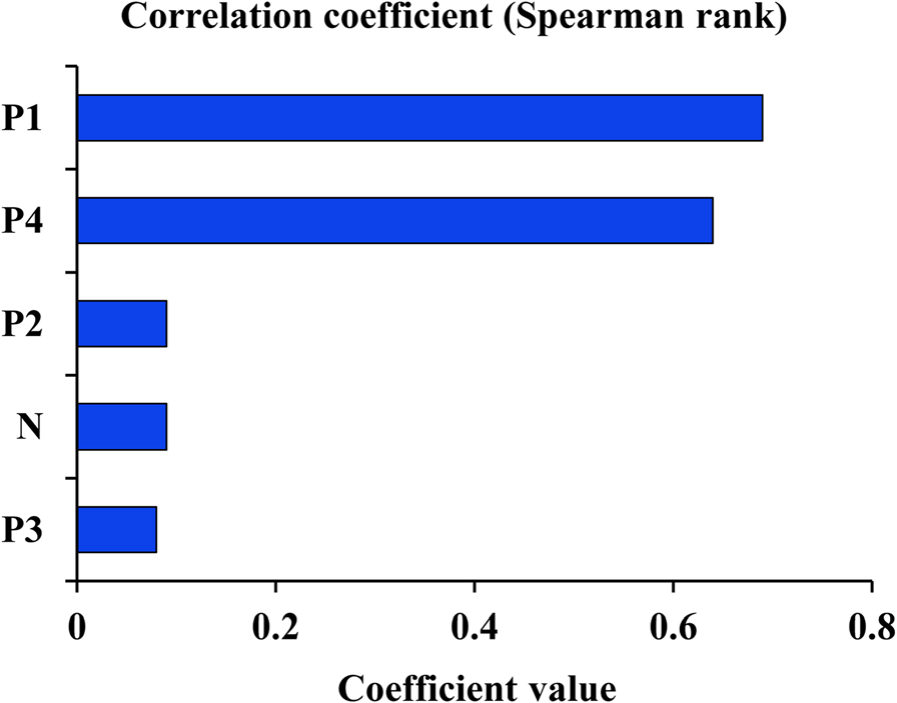
Correlation sensitivity analysis of the model. N: Number of cattle exported from Russia every year, P1: Probability of occurrence of FMD outbreaks in the Russian surveillance zone, P2: Probability of safe cattle with suspected FMD infection FMD in the Russian surveillance zone, P3: Probability that cattle are not protected after vaccination, P4: Probability of infected cattle not detected by ELISA

The sensitivity correlation analysis revealed that the probability of occurrence of FMD outbreaks in the Russian surveillance zone (P1) had a greater impact on Russian exports of FMD positive cattle (Fig. 4). ELISA on bovine detection sensitivity (P4) had the second highest impact. The probability of safe cattle with suspected FMD infection FMD in the Russian surveillance zone (P2) and the number of cattle exported from Russia every year (N) had little influence on the output of the model. Compared with other variables in the model, the probability that cattle are not protected after vaccination (P3) had the least impact on the model output.

### Semi-quantitative model based on sensitivity analysis to assess the risk of flaws existed in the defense measures system in Russia

In the stochastic decision tree model, the probability of cattle infected FMD in the surveillance zones of Russian was 1.29 × 10^− 6^. Based on the sensitivity analysis, it was found that the biggest influence on cattle infection with FMD was the probability of cattle infected FMD in the surveillance zones of Russian. Hence, a semi-quantitative model was constructed to assess the risk of defensive deficiencies in the existing Russian detection zones. After 10,000 iterations of the Monte Carlo simulations, probability distributions produced by each node (first-class index) and the risk probability of measures (P) were obtained. The probability of the risk for measures to control FMD in Russia was 1.84 × 10^− 5^ (Table 3).

**Table 3:** Results showing inputs and outputs for the risk of FMD prevention and control system from Russia

Probability	Minimum	Mean	Maximum
**Input probabilities**			
PA	0.017	0.063	0.350
PB	0.266	0.334	0.405
PC	0.402	0.500	0.593
PD	0.001	0.021	0.042
PE	0.022	0.082	0.142
**Outputs**:			
P	3.37 × 10^− 7^	1.84 × 10^− 5^	9.38 × 10^− 5^

### Spatial analysis for FMD outbreaks surveillance

**Fig. 5:**
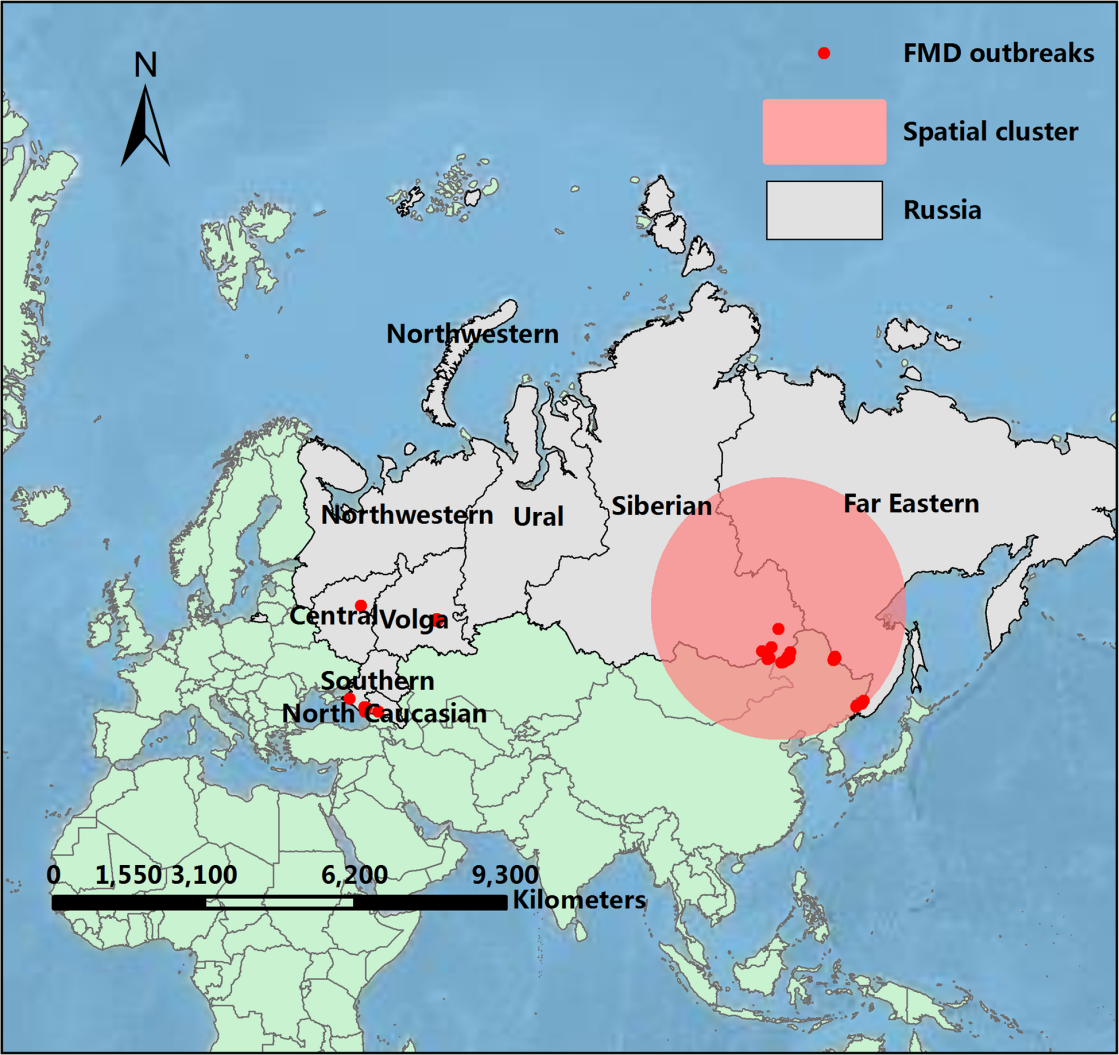
Geographical localization of FMD clusters in Russia. The maps were generated with QGIS Version 2.18 (https://www.qgis.org/en/site/index.html) and the base map was Bing Maps from QuickMapServices

After conducting a risk quantification of exported cattle and defense measures system in Russia, we attempted to explore some potential key points about the exact division of the FMD outbreak area and establish a precise and comprehensive surveillance of the zones involved. Spatial scan statistics were proposed to analyze the FMD outbreaks and identify the surveillance zones that were significant to focus on in order to prevent and control these outbreaks. The results showed that the spatial scan statistics including 47 outbreak zones as certain infection sources for the animals in Russia. One space-time cluster was identified which has persisted for 5 years. The most likely cluster (P < 0.01) was found to be in the Eastern and Siberian Central regions, which consisted of 27 districts. This space-time cluster existed from July 5, 2010, to February 12, 2014, with 1877 observed FMD cases. The space-time cluster is shown in Fig. 5.

## Discussion

FMD is a severe disease listed by OIE, which has established an official list of free countries and zones [21]. Outbreaks from disease-endemic regions continuously threaten livestock industries [22–24]. After analyzing outbreaks of FMD in various countries, studies have indicated that the virus is widely prevalent in agricultural countries, such as China and Mongolia [25, 26]. FMD had a worldwide distribution in the past, concentrated in Asia, Africa and the parts of Europe adjacent to Asia [27, 28]. After preventing and controlling the disease via international organizations (such as the FAO and OIE), FMD has shifted from a widespread worldwide distribution to a localized regional distribution [29]. In recent years, FMD has completely disappeared in Europe and North America. Additionally, sixty-six countries or regions have been accredited by OIE as non-FMD countries or regions [30]. There are fewer outbreaks of FMD in Europe than in other continents worldwide, though some countries of southern Europe continue to have outbreaks, which threaten major livestock-rearing countries (1991) [31]. Recently, there have been continuous FMD outbreaks in China (January 2017), Mongolia (February 2017), Zimbabwe (June 2017) and the Russian Federation (October 2017) [32]. According to the outbreak data of FMD provided by the FAO [33], there have been 71 outbreaks of FMD in Europe since 2005. FMD has occurred mainly in Russia and Bulgaria. Russia represents the largest portion of European outbreaks. In this study, a four-level stochastic decision tree risk model was constructed to assess the occurrence of FMD in surveillance areas in Russia. The results derived from these stochastic decision tree models showed that there was low risk in the surveillance zones for cattle infected with FMD, and the risk was reduced by taking appropriate risk management measures. Due to the limited amount of data available, the model used only simple distributions for simulation. Other data related to FMD outbreaks, such as FMD outbreaks in non-monitored areas and FMD in wild animals, were not used. However, the risk estimates provided by our study could be improved by incorporating future monitoring results to make the results more accurate. According to the sensitivity analysis, the outbreak probability of FMD in the monitored area of Russia had the greatest influence on the output results of the model. Therefore, we can reduce the possibility of FMDV infection in exported cattle by improving the prevention and control measures of FMD. Furthermore, Monte Carlo simulations with its advantages of simulating the characteristics of random events have been widely used in the decision tree assessment of the risk of FMD [34]. However, there are some disadvantages of Monte Carlo simulation, for example, an entirely new simulation must be executed each time. If a parameter changes, it may be time-consuming when the desired accuracy is high [35]. These disadvantages of Monte Carlo simulation may cause errors in node simulation sampling and affect the results of the risk assessment.

Based on the semi-quantitative model of sensitivity analysis, the risk analysis of FMD prevention and control measures was carried out to further study the causes of the FMD outbreak. Semi-quantitative models will also assist in the selection of risk priorities when risk measures need to be improved. Russia has taken some measures for the prevention and control of FMD in terms of some aspects including regional management, immunization, monitoring, animal and product flow control, and emergency treatment [27, 36]. To ensure animals are free from FMD, restrictive quarantine measures were taken at the international ports and stations, and immunization injections were strengthened in the border and coastline areas. These defense measures have protected animal husbandry to a large extent. However, there are still some problems in measures that increase the risk for animals affected by FMD. For example, culling measures have hidden troubles, and non-immune zone monitoring is often inadequate. In addition to this, in the immunized zone, Russia immunized cattle, sheep, and goats, while pigs and wildlife are ignored. These control failure in the defense system that are meant to prevent and control the FMD outbreaks may be the main reasons for cattle infected with FMD to be exported from Russia. Besides, if the number of under-reporting of FMD is in Russia, this will also bring serious results. The number of outbreaks in Russia is under-reported, which not only conceals the actual epidemic situation of animal epidemics, affects the accurate judgment of OIE, FAO and governments, but also hinders relevant organizations, institutions and countries to take timely risk reduction and control measures. Measures to control FMD in Russia need to be further explored based on existing factors.

Early assessment of disease cluster regions is an essential part of the surveillance of diseases [37]. The spatial time scan statistic is a spatial analysis tool that explores whether there is an aggregation of disease in time, space, or space-time, and tests whether the disease occurs randomly in time or space. This method has a role in the early warning and monitoring of disease outbreaks and can provide advice for the separation of the FMD outbreak area. In order to understand the spatial epidemiology of FMD outbreaks in Russia, the spatial analysis was found to contribute strongly to elucidating the distribution and aggregation of outbreak zones with cases of FMD. After analyzing FMD outbreaks data from 2006 to 2017 by spatial scan statistics, the cluster regions were found to be in the Eastern and Siberian Central regions of Russia, while the cluster time was found to be during 2010/7/5 ~ 2014/2/12 (Fig. 5**)**. Although the significant spatial correlation is mainly concentrated in 2010 ~ 2014, it is still necessary to strengthen monitoring in the Far Eastern and Siberian Central regions during the other periods. The spatial cluster was probably due to problems in prevention and control measures and the temperature during that time being suitable for virus transmission, or to other factors. Further research on the distribution of FMD could be carried out in the future by adding spatiotemporal geographic data. The spatial time scan statistic makes up the deficiency of quantitative and semi-quantitative analysis in time and space. To help policymakers analyze the spatial and temporal clusters of FMD to design effective interventions.

The combination of various analyses provided a clue for agricultural countries to prevent FMD spreading into their livestock, increasing the accuracy of regional divisions in FMD surveillance zones and strengthening the FMD integrated surveillance in a general approach. Therefore, the exporting country can establish a disease early warning system to guarantee the international exchange of animal product safety. The risk analysis model of epidemic disease can guide epidemic prevention and control. However, these models require professional software to interpret and explain. Hence, some experimental investigators cannot apply these models directly. Some simple and friendly software or online systems should be provided. In the future, an online prediction server will be constructed based on the model in this study. It can be used to store and check the annual FMD outbreak data and predict the risk of future outbreaks, which provides good suggestions for the prevention and control of FMD.

## Conclusions

In this work, we constructed a stochastic decision tree model to provide a preliminary estimate of the risk of FMD positive cattle being exported from Russia. The results showed that there was a risk of FMDV among cattle exported from Russia, and the sensitivity analysis indicated that the infection rate of cattle in the monitored area was the key factor. Then, a semi-quantitative model was constructed to assess the risk of existing flaws in the defense system in Russia by grading the FMD defense measures employed. Since stochastic decision tree and semi-quantitative analysis cannot accurately analyze the geographical distribution of the epidemic, we also constructed a retrospective permutation space-time scan model to analyze whether there was a spatial-temporal cluster distribution for FMD. The results also showed that the combination of the Monte Carlo simulation and the space-time scan model can better help agricultural countries to prevent FMD. Policymakers can use the results of risk analysis to develop targeted surveillance plans and preventive measures to improve national capacity for early detection of animal diseases, thereby reducing the risk of the trade.

## Methods

### Data preparation

In the stochastic decision tree, the number of cattle exported from Russia every year (N) was obtained from the China Industrial Information Network [38] (see supplementary materials, S1 Table). FMD outbreaks in Russia from 2005 to 2017 (P1) were collected from the World Organization for Animal Health (OIE) [39] (see supplementary materials, S2 Table). Sampling numbers of cattle infected with FMD were collected from the results of cattle being monitored for antibodies to FMDV using structural polyproteins in 2015 (P2) (see supplementary materials, S3 Table). Samples from vaccinated animals infected with FMD were taken from the testing results of antibodies to FMDV non-structural polyproteins in 2013 (P3) (see supplementary materials, S4 Table). All data for nodes P2 and P3 were collected from the Russian Federation Service for Veterinary and Phytosanitary Surveillance. The minimum and maximum values for diagnostic sensitivity were taken from a study of virus detection using ELISA (P4) [40, 41]. Referring to the international standards (AS/NZS ISO 31,000:2009 Risk management - principles and guidelines), combined with the risk identification, analysis and evaluation methods and risk management process proposed in Chinese national standards (GB/T27921-2011 Risk management - Risk assessment techniques), a semi-quantitative model of FMD positive cattle export caused by the failure of Russian control measures was established. Materials regarding the measures used in the prevention and control of FMD were taken from a technology conference (Ministry of agriculture and FAO signed memorandum of cooperation on further strengthening animal disease prevention and control, Paris, France, 2013) [42] and Russian Federation Service for Veterinary and Phytosanitary Surveillance. The localities of FMD cases in Russia from 2006 to 2017 were collected from the Food and Agricultural Organization of the United Nations (FAO) [43] (see supplementary materials, S5 Table).

### Monte Carlo simulation for a stochastic decision tree assessment model

**Fig. 6:**
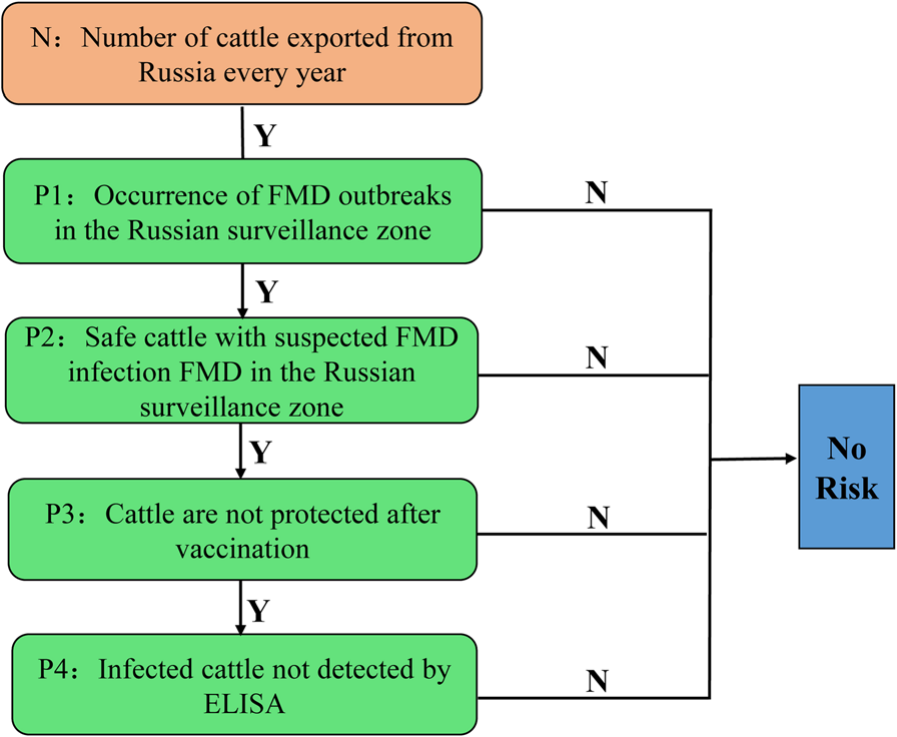
Stochastic decision tree for the quantitative risk assessment model

In this work, a stochastic decision tree assessment model was constructed based on the Monte Carlo stochastic simulation (Fig. 6.). The model was developed in the MATLAB environment, version 7.11.0. Monte Carlo simulations were run for 10,000 iterations to produce probability distributions for the occurrence of the hazard at each node [44]. Subsequently, to assess the specific impact of model variables on the output value, a sensitivity analysis chart was developed showing graphically the degree of influence of each variable.

### Definition of distributions for each node from the stochastic decision tree

#### Stochastic decision tree assessment model for FMD outbreaks in the surveillance zone

**Fig. 7:**
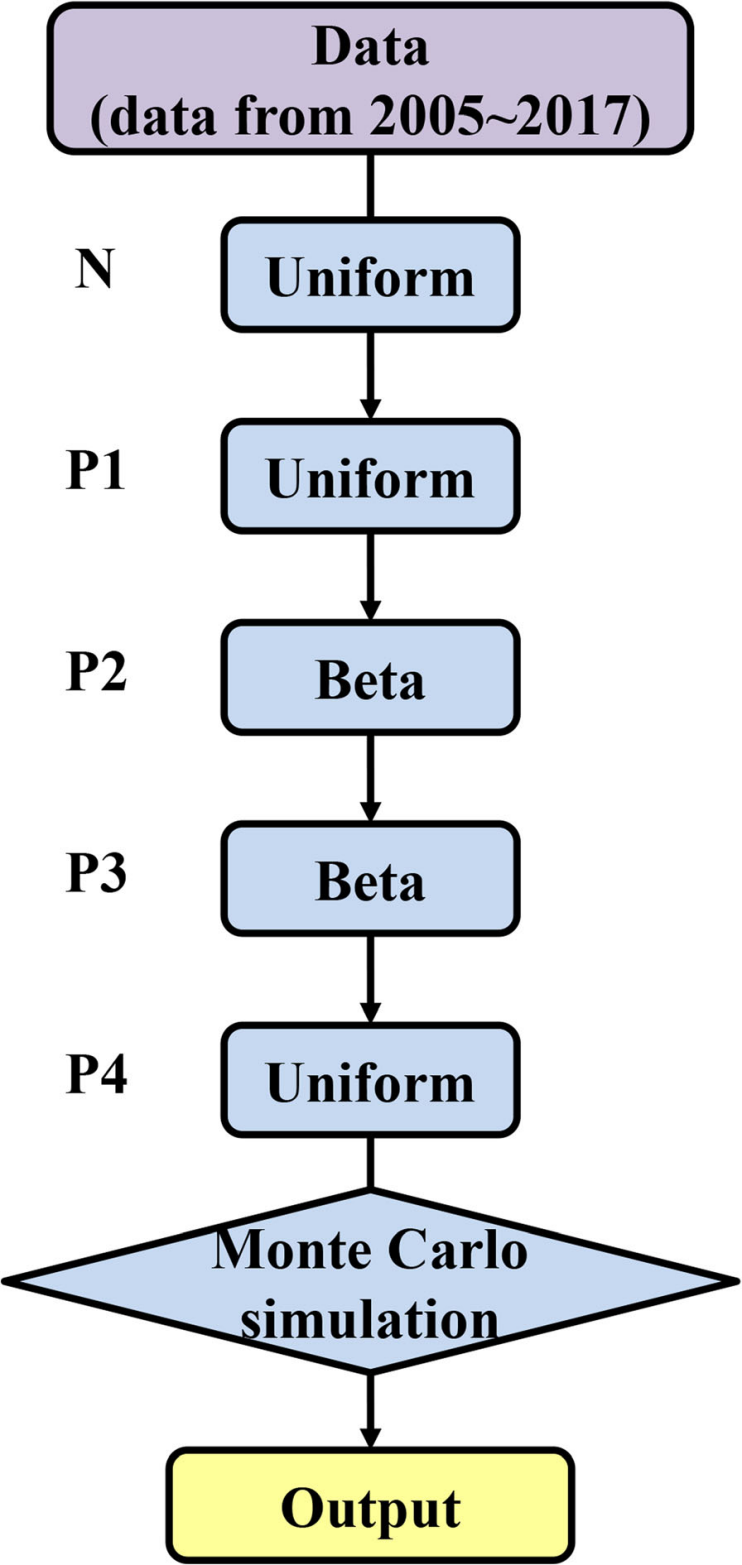
Distributed nodes of risk assessment model

No epidemic disease occurred in a designated area within the prescribed time limit. Surrounding this area, according to the natural environment, geographical conditions and disease types, the buffer zone is called the surveillance zone. The surveillance area has an advanced epidemic surveillance plan. A stochastic decision tree model was used to evaluate the risk of FMD outbreak in the surveillance zone. P in P1 to P4 represents the calculated probability of this node, and Arabic numerals represent the order of nodes. When the data distribution is not clear, it can be replaced by a simple distribution. The beta distribution is often used to analyze the probability density distribution of the probability of an event [45]. Therefore, in the stochastic decision tree model, for the nodes with uncertain distribution, we prefer to use the beta distribution to build the model (Fig. 7.).

##### N: Number of cattle exported from Russia every year

The number of cattle exported from Russia every year was calculated from the forecast of supply and demand balance of the Russian beef market (see supplementary materials, S1 Table). The expected number of cattle (N) exported from Russia was modeled using a uniform distribution with parameters ${a}_{y}$(minimum number of cattle) and ${b}_{y}$ (maximum number of cattle) which were calculated by using ${S}_{y}$, ${P}_{y}$ and ${E}_{y}$.

1 $${a}_{y}\left({b}_{y}\right)=\frac{{S}_{y}}{{P}_{y}}\times {E}_{y}$$

where ${S}_{y}$ is the number of cattle slaughtered and ${P}_{y}$ is the total beef production from Russia in a particular year. ${E}_{y}$ is the number of beef exports from Russia in a particular year. Thus, values of a_y_ and b_y_ are

$${a}_{2011}=\frac{\text{6,720,000}}{\text{1360}}\times 8=39,530$$

$${ b}_{2017}=\frac{\text{6,853,000}}{\text{1355}}\times 10=50,576$$

##### P1: Probability of occurrence of FMD outbreaks in the Russian surveillance zone

The outbreak data from 2005 to 2017 were used to analyze the possibility of FMD outbreaks in Russia. According to S2 Table of supplementary materials, the minimum probability of FMD outbreak in Russia is 0, and the maximum probability is 0.333. Therefore, the uniform distribution of minimum and maximum were used to determine the possibility of an FMD outbreak in Russia.

##### P2: Probability of safe cattle with suspected FMD infection in the Russian surveillance zone

Some major surveillance zones can become safe, free zones for FMD after years of prevention and control measures. Animals in these free zones are inclined to be unvaccinated. However, due to the variety of ways in which FMD is transmitted, unvaccinated animals are most likely to be infected, even if the animal is located in a safe area of low risk. Unvaccinated cattle will produce antibodies to structural proteins after being infected with FMDV, so we used the detection results of FMD structural multi-protein antibodies in cattle for modeling. The probability of unvaccinated animals becoming infected with FMD was estimated using a beta distribution (α1e, α2e).

2 $${{\upalpha }1}_{e}={\text{N}\text{P}}_{e}+1$$

3 $${{\upalpha }2}_{e}={\text{N}\text{T}}_{e}-{\text{N}\text{P}}_{e}+1$$

Where ${\text{N}\text{P}}_{e}$(70) denoted the number of positive cases detected by ELISA in Russia in 2017, and ${\text{N}\text{T}}_{e}$(4449) is the total number of tested animals.

##### P3: Probability that cattle are not protected after vaccination

Vaccination is a very common measure for the prevention and control of FMD. However, some animals are not protected after vaccination. This may be due to the fact that the animal antibody is not produced or the concentration is low after the vaccine, which cannot be protected immediately, so that the animal may still be infected with the disease. Or there are problems in the process of vaccine processing and storage, making the vaccine itself ineffective. Animals successfully vaccinated do not produce non-structural protein (NSP). Based on the materials available in Russia, the probability of detection of NSP in unprotected cattle after vaccination was modeled as a beta distribution (α1_v_, α2_v_). ${{\upalpha }1}_{v}={\text{N}\text{P}}_{v}+1$, ${{\upalpha }2}_{v}={\text{N}\text{T}}_{v}-{\text{N}\text{P}}_{v}+1$ with ${\text{N}\text{P}}_{v}$ (102) denoting the number of positive cases in Russia and ${\text{N}\text{T}}_{v}$ (17,190) is the total number of detected animals.

##### P4: Probability of infected cattle not detected by ELISA

The primary diagnosis of FMD can be made by the clinical manifestations and pathological changes. Among the several diagnostic methods, ELISA has the advantage of using inactivated antigens and has been regarded as the prescribed test for international trade by the OIE. The specificity and sensitivity of ELISA are different when tested by using different reagents. The minimum and maximum values of diagnostic sensitivity were 84.0 and 99.7 %, respectively.

Table 4 summarizes the notation, variable description, and sources of information used to formulate and parameterize a model to estimate the values of parameters of the model.

**Table 4:** Notation, variable description, and sources of information used to formulate and parameterize a model to assess the risk of FMD exported from Russia

Notation	Variable description	Parameterization	Source of information
**N**	Number of cattle exported from Russia every year	Uniform (${a}_{y},{b}_{y}$)	China Industrial Information Network
y	FMD outbreaks year	NA	Model equations
${a}_{y}$	The minimum number of exported cattle (n)	$\frac{{S}_{y}}{{P}_{y}}\times {E}_{y}$	Model equations
${b}_{y}$	The maximum number of exported cattle (n)	$\frac{{S}_{y}}{{P}_{y}}\times {E}_{y}$	Model equations
${S}_{y}$	The number of cattle slaughtered (n)	NA	S1 Table
${P}_{y}$	Total beef production in Russia (kt)	NA	S1 Table
${E}_{y}$	Amounts of beef exports from Russia (kt)	NA	S1 Table
**P1**	Probability of occurrence of FMD outbreaks in the Russian surveillance zone	Uniform (${a}_{1},{b}_{1}$)	OIE (S2 Table)
${a}_{1}$	The minimum outbreak to total outbreaks over a certain period	minimum outbreak/ total outbreaks	Model equations
${\text{b}}_{1}$	The maximum outbreak to total outbreaks over a certain period	maximum outbreak/ total outbreaks	Model equations
**P2**	Probability of safe cattle with suspected FMD infection FMD in the Russian surveillance zone	Beta (${{\upalpha }1}_{e},{{\upalpha }2}_{e}$)	S3 Table
${NP}_{e}$	Number of positive cases detected by Elisa	NA	S3 Table
${NT}_{e}$	Total number of detected animals	NA	S3 Table
${{\upalpha }1}_{e}$	Parameter in beta distribution	${\text{N}\text{P}}_{e}+1$	Model equations
${{\upalpha }2}_{e}$	Parameter in beta distribution	${\text{N}\text{T}}_{e}-{\text{N}\text{P}}_{e}+1$	Model equations
**P3**	Probability that cattle are not protected after vaccination	Beta (${{\upalpha }1}_{v},{{\upalpha }2}_{v}$)	S4 Table
${\text{N}\text{P}}_{v}$	Number of positive cases in vaccinated animals	NA	S4 Table
${\text{N}\text{T}}_{v}$	Total number of vaccinated animals	NA	S4 Table
${{\upalpha }1}_{v}$	Parameter in beta distribution	${\text{N}\text{P}}_{v}+1$	Model equations
${{\upalpha }2}_{v}$	Parameter in beta distribution	${\text{N}\text{T}}_{v}-{\text{N}\text{P}}_{v}+1$	Model equations
**P4**	Probability of infected cattle not detected by ELISA	Uniform (${a}_{4},{b}_{4}$)	Documentation
${a}_{4}$	The minimum probability of infected cattle not detected by ELISA	1- maximum values diagnostic sensitivity	Model equations
${b}_{4}$	The maximum probability of infected cattle not detected by ELISA	1- minimum values diagnostic sensitivity	Model equations

#### Semi - quantitative risk assessment model of defense system

We hypothesis that Russian cattle were infected with FMD due to the control failure of the FMD defenses system as defensive measures were not strictly enforced by local farms. Then, we try to identify the measures to control FMD and analyze them with 5 first-class indices (PA: Regional management; PB: Immune protection; PC: Defense surveillance; PD: Control of animal and product flow; PE: Emergency treatment). The first-class indices of each control measure is refined into the second level index for a more detailed description (PA1:The accuracy of the regional division; PA2: Reliability of Regionalization of FMD; PA3: The effectiveness of regional management; PB1: Immune coverage; PB2: The effectiveness of the FMD vaccine; PB3: Immune comprehensive of susceptible animals; PC1: Epidemiological sampling principle; PC2: The comprehensive of susceptible animal infection monitoring; PC3: Surveillance of wild animals; PD1: Illegal transport of animals and products; PD2: The probability of transporting infected animals between different regions; PD3: The validity of the rules for the transport of animals and products; PE1: Culling measures of diseased animals; PE2: The rigor of the principles of emergency treatment of FMD; PE3: Treatment of slaughtered meat in infected zone) **(**Table 5**)**. The relevant risks of each secondary indicator are quantified into seven intervals (see supplementary materials, S6 Table). Evaluation indicators were assigned a risk spectrum after determination of the risk level of defensive measures by risk analysis experts (Bing Niu and Qin Chen from Shanghai University, Jianhua Xiao from Northeast Agricultural University, Qiang Zhang from Plant and Food Inspection and Quarantine of Shanghai Customs, and Quan Wang from Shanghai Veterinary Research Institute). The risk spectrum of each evaluation indicator represented a uniform distribution with parameters of a minimum and maximum score **(**Table 5**)**. Each evaluation indicator, weighted from 1 to 3, was assigned by risk analysis experts. After that, a semi-quantitative risk assessment model of FMD was built to evaluate the impact of the control failures on the prevention and control system.

**Table 5:** Parameters of FMD defense measures risk assessment in Russia

First-class index	Second-class index	Weight	Uniform	
			Min	Max
PA (Regional management)	PA1 The accuracy of the regional division	2	0.05	0.3
	PA2 Reliability of Regionalization of FMD	1	0.001	0.05
	PA3 The effectiveness of regional management	3	1 × 10^− 6^	0.001
PB (Immune protection)	PB1 Immune coverage	3	0.5	0.7
	PB2 The effectiveness of the FMD vaccine	3	0.001	0.05
	PB3 Immune comprehensive of susceptible animals	2	0.3	0.5
PC (Defense surveillance)	PC1 Epidemiological sampling principle	1	0.5	0.7
	PC2 The comprehensive of susceptible animal infection monitoring	3	0.3	0.5
	PC3 Surveillance of wild animals	2	0.5	0.7
PD (Control of animal and product flow)	PD1 Illegal transport of animals and products	2	0.001	0.05
	PD2 The probability of transporting infected animals between different regions	3	0.001	0.05
	PD3 The validity of the rules for the transport of animals and products	1	1 × 10^− 6^	0.001
PE (Emergency treatment)	PE1 Culling measures of diseased animals	3	0.05	0.3
	PE2 The rigor of the principles of emergency treatment of FMD	2	0.001	0.05
	PE3 Treatment of slaughtered meat in infected zone	3	0.001	0.05

### Model formulation

#### Output calculation in the stochastic decision tree assessment model

The results of outputs were calculated based on different distribution parameters from each node in MATLAB, and using Monte Carlo simulations at 10,000 iterations. The risk probability of cattle infected with FMD in the surveillance zones of Russia was considered to be a product of the probability distributions ($\text{p}=\text{p}1\times \text{p}2\times \text{p}3\times \text{p}4$). The probability of at least one FMD positive case exported from Russia per year was estimated as ($\text{q}=1\text{-}{\left(1-p\right)}^{N}$). The predicted number of positive cattle exported from Russia per year was assumed to be ($\text{e}=\text{N}\times \text{p}$) [40]. Based on the Spearman rank correlation coefficient, the sensitivity analysis was carried out by using rank correlation. Through analysis, the rank correlation coefficient between the selected output variable and the sample of each input distribution was calculated. The higher the correlation between input and output, the more significant the decisive effect of input on output value.

#### Output calculation in the semi - quantitative risk assessment model

According to the score sheet of the measures to control FMD, each measure was assigned a risk value which was graded by experts. The probabilities of the 5 first-class indices (PA-PE) were described as:

6 $$\text{P}\left(X\right)=\frac{\sum P{X}_{n}{W}_{{X}_{n}}}{\sum {W}_{{X}_{n}}}$$

Where ${X}_{n}$ is the second-class index of each first-class index, and ${W}_{{X}_{n}}$ is the weight of each evaluation indicator, ranging from 1 to 3. X represents A, B or C, and n represents 1, 2 or 3. The probability of the risk for measures to control FMD in Russia was estimated as $\text{P}=\text{P}\text{A}\times \text{P}\text{B}\times \text{P}\text{C}\times \text{P}\text{D}\times \text{P}\text{E}$. The semi-quantitative risk assessment model was developed in the MATLAB environment, version 7.11.0.

### Spatial analysis

The retrospective permutation space-time scan model was developed in the SaTScan environment. This method consisted of building a space-time cylinder to scan the study area by placing a number of circles (spatial windows) [46, 47]. The radius at the bottom of the cylinder represented the geographical position and size of the cluster area. The height of the cylinder denoted the date of the outbreaks [48]. With constant changes in radius and time, the spatial window changed dynamically, and the number of positive and/or negative events that occurred can be counted. In this study, we described the geographical and temporal occurrence of FMD and analyzed the data for spatial and temporal clusters. With the setting of “day” for time precision, the model utilized FMD cases in order to define the scanning window with a study period of 2006/1/1- 2017/12/31.

## Supplementary Information

**Additional file 1: S1 Table.** Summary of Russian beef market supply and demand from 2011 to 2022.

**Additional file 2: S2 Table.** FMD outbreaks in Russia from 2005 to 2017 from OIE.

**Additional file 3: S3 Table. **Results of Cattle Monitoring for Antibodies to FMD Structural Polyprotein in Russia without FMD.  

**Additional file 4: S4 Table.** Results of cattle monitoring for antibodies to FMDV non-structural polyproteins in the RF.  

**Additional file 5: S5 Table.** FMD outbreaks in Russia from 2006 to 2017.

**Additional file 6: S6 Table.** Rating scale of Foot-and-mouth disease prevention and control in Russia.

## Data Availability

The datasets used and/or analyzed during the current study are available from the corresponding author on reasonable request.

## Abbreviations

FMD: Foot-and-mouth disease
FMDV: Foot-and-mouth disease virus
NSP: non-structural protein
OIE: World Organization for Animal Health
FAO: Food and Agricultural Organization of the United Nations
